# Supplementary material for: Unveiling the Hidden Causes: Identifying the Drivers of Human–Elephant Conflict in Nilgiri Biosphere Reserve, Western Ghats, Southern India
Source: Animals (Basel). 2024 Nov 7;14(22):3193. doi: 10.3390/ani14223193 (PMC11590974; doi:10.3390/ani14223193)
Supplement: Supplementary file 1 [file animals-14-03193-s001.zip › animals-3218988-supplementary.pdf]

S-Figure 1: Photographs showing grass growth in the deciduous forest of Nilgiri Biosphere Reserve, Western Ghats, India.

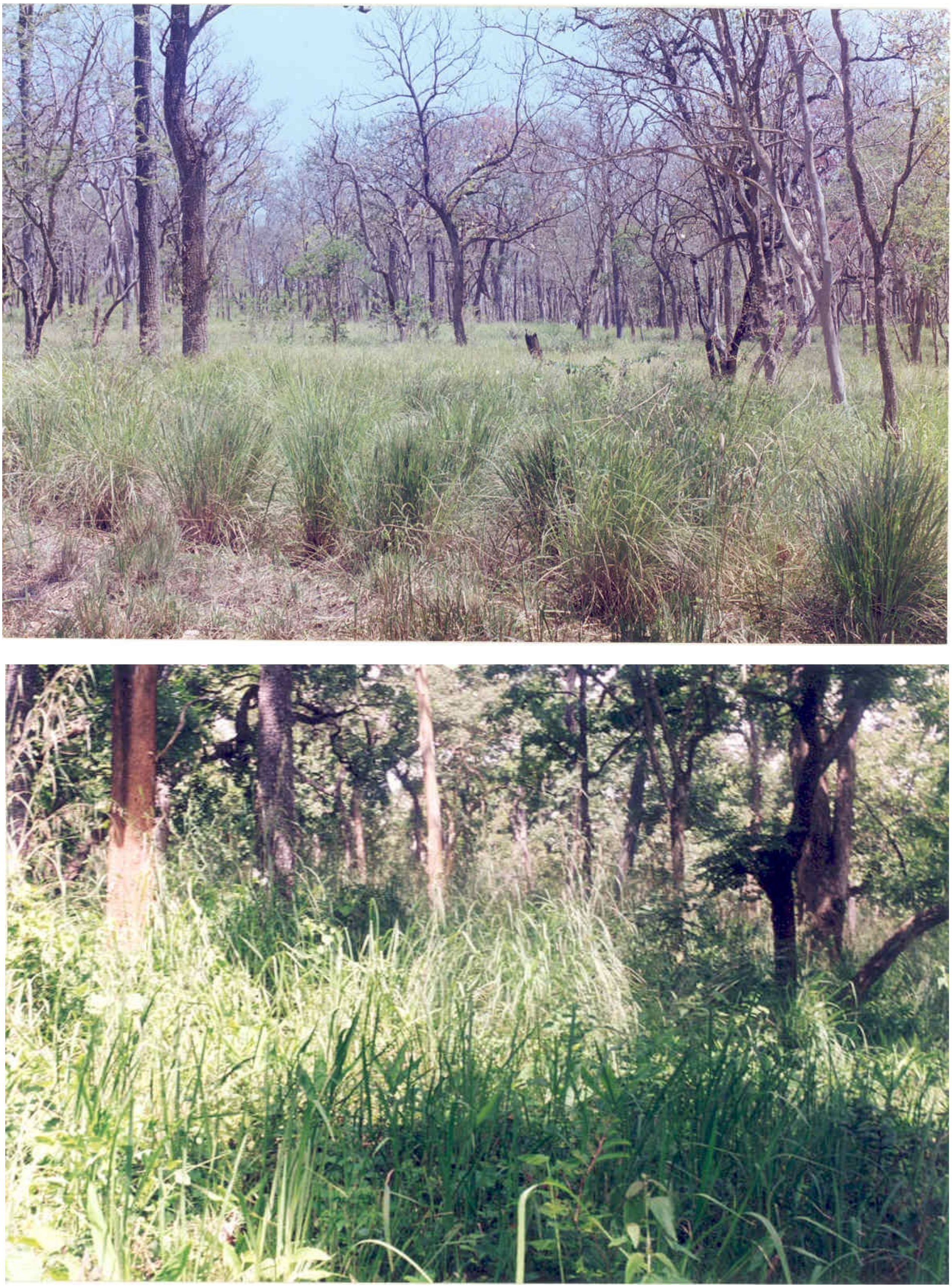

S-Table 1: HEC level in relation various covariates recorded in Nilgiri Biosphere Reserve, Western Ghats, India.

**Covariates**	**Category**	**Crop damage by elephant**	**Property damage by elephant**	**Human casualty by elephant**	**Elephant mortality by electrocution**
Rainfall (mm)	Low (< 1000)	10.3 ± 1.99	0.1 ± 0.06	0.7 ± 0.15	0.6 ± 0.18
	High (> 1000)	32.9 ± 4.26	1.9 ± 0.46	1.9 ± 0.29	0.3 ± 0.07
	MWU (p-value)	1710 (0.066)	**1619 (0.004)**	1778 (0.106)	2382 (0.134)
Ambient temperature (^{0}C)	Low (< 28)	25.9 ± 4.06	1.6 ± 0.4	1.3 ± 0.23	0.4 ± 0.09
	High (> 28)	30.2 ± 5.75	1.3 ± 0.71	2.2 ± 0.48	0.4 ± 0.11
	MWU (p-value)	**1901 (0.042)**	2584 (0.302)	**1841 (0.016)**	2311 (0.654)
Humidity (%)	Low (< 70)	12.1 ± 3.38	0 ± 0	0.8 ± 0.22	0.3 ± 0.13
	High (> 70)	28.5 ± 3.57	1.6 ± 0.38	1.6 ± 0.24	0.4 ± 0.08
	MWU (p-value)	846 (0.905)	**606 (0.042)**	786 (0.755)	831 (0.981)
Grass height (cm)	Low (< 13)	47 ± 6.58	3.1 ± 0.81	2.8 ± 0.48	0.3 ± 0.11
	High (> 13)	14 ± 2.59	0.4 ± 0.12	0.7 ± 0.13	0.4 ± 0.09
	MWU (p-value)	**3871 (<0.000)**	**3319 (0.002)**	**3686 (<0.000)**	2633 (0.725)
Grass cover (%)	Low (< 70)	0.2 ± 0.14	0.3 ± 0.18	0.5 ± 0.18	1.1 ± 0.26
	High (> 70)	33.9 ± 3.91	1.8 ± 0.43	1.8 ± 0.27	0.2 ± 0.05
	MWU (p-value)	**352 (<0.000)**	1519 (0.081)	**1229 (0.004)**	**2509 (<0.000)**
Grass soft texture (%)	Low (< 50)	53.8 ± 6.99	0.5 ± 0.14	2.4 ± 0.46	0.2 ± 0.05
	High (> 50)	12.2 ± 2.26	3.3 ± 0.89	1.1 ± 0.21	0.5 ± 0.1
	MWU (p-value)	**4367 (<0.000)**	**3198 (0.002)**	**3472 (<0.000)**	**2226 (0.050)**
Grass hard texture (%)	Low (< 13)	12.2 ± 2.26	3.3 ± 0.89	1.1 ± 0.21	0.5 ± 0.1
	High (> 13)	53.8 ± 6.99	0.5 ± 0.14	2.4 ± 0.46	0.2 ± 0.05
	MWU (p-value)	**817 (<0.000)**	**1986 (0.002)**	**1713 (<0.000)**	**2958 (0.050)**
Green grass (%)	Low (< 50)	39.4 ± 5.19	2.2 ± 0.59	1.8 ± 0.31	0.2 ± 0.06
	High (> 50)	11.6 ± 2.61	0.6 ± 0.2	1.2 ± 0.3	0.6 ± 0.14
	MWU (p-value)	**4262 (<0.000)**	3096 (0.105)	**3431 (0.008)**	**2400 (0.054)**
Dry grass (%)	Low (< 25)	30.5 ± 5.28	2.3 ± 0.6	2.1 ± 0.37	0.5 ± 0.11
	High (> 25)	22.9 ± 3.39	0.4 ± 0.14	0.9 ± 0.15	0.2 ± 0.07
	MWU (p-value)	**2282 (0.059)**	3144 (0.063)	3039 (0.280)	3115 (0.076)
Reproductive phase (%)	Low (< 12)	16.1 ± 3.48	0.4 ± 0.16	0.6 ± 0.16	0.3 ± 0.1
	High (> 12)	33.4 ± 4.69	2.1 ± 0.53	2.1 ± 0.32	0.4 ± 0.1
	MWU (p-value)	2187 (0.106)	**2216 (0.052)**	**1689 (<0.000)**	2345 (0.186)
Semi-evergreen / evergreen habitat	Low (< 20)	24.2 ± 3.88	1.2 ± 0.32	1.1 ± 0.2	0.5 ± 0.1
	High (> 20)	32.5 ± 6.09	2 ± 0.79	1.8 ± 0.32	0.1 ± 0.04
	MWU (p-value)	2207 (0.125)	2582 (0.959)	2666 (0.760)	**3096 (0.007)**
Tropical-deciduous habitat	Low (< 50)	34.4 ± 5.26	2.2 ± 0.56	1.0 ± 0.17	0.2 ± 0.06
	High (> 50)	22.4 ± 4.22	0.3 ± 0.12	1.9 ± 0.34	0.6 ± 0.14
	MWU (p-value)	**3349 (0.010)**	**3178 (0.015)**	2802 (0.677)	**2186 (0.007)**
Tropical dry-	Low (< 50)	29.3 ± 3.65	2.6 ± 0.69	1.5 ± 0.26	0.2 ± 0.07

thorn habitat	High (> 50)	11.8 ± 35.9	0.4 ± 0.11	1.6 ± 0.36	0.5 ± 0.12
	MWU (p-value)	**1639 (0.010)**	**3261 (0.024)**	2500 (0.215)	**2393 (0.033)**
Hum settlement / cultivation	Low (< 25)	23.4 ± 3.64	1.2 ± 0.39	1.2 ± 0.2	0.3 ± 0.08
	High (> 25)	33.8 ± 6.52	2.5 ± 0.76	1.8 ± 0.32	0.5 ± 0.13
	MWU (p-value)	2224 (0.143)	**1517 (0.002)**	2570 (0.926)	2307 (0.128)
Waterbody area	Low (< 5)	27.3 ± 3.94	1.6 ± 0.43	1.6 ± 0.27	0.3 ± 0.08
	High (> 5)	26.6 ± 5.31	1 ± 0.32	1.4 ± 0.24	0.5 ± 0.18
	MWU (p-value)	1507 (0.162)	1684 (0.473)	1508 (0.142)	1680 (0.440)
Elephant population size (Total No. of individuals)	Low (< 200)	66.7 ± 7.56	4.4 ± 1.1	3.9 ± 0.6	0.2 ± 0.06
	High (> 200)	11.8 ± 2.21	0.3 ± 0.1	0.6 ± 0.11	0.4 ± 0.09
	MWU (p-value)	**4171 (<0.000)**	**3220 (<0.000)**	**3840 (<0.000)**	2106 (0.355)
Elephant density (No. / km^2)	Low (< 0.5)	39.7 ± 4.36	2.1 ± 0.5	2.1 ± 0.3	0.2 ± 0.05
	High (> 0.5)	0.5 ± 0.21	0.2 ± 0.11	0.3 ± 0.12	0.7 ± 0.18
	MWU (p-value)	**4587 (<0.000)**	**3063 (0.001)**	**3712 (<0.000)**	**2046 (0.027)**
Adult male %	Low (< 5)	11.8 ± 2.66	0.4 ± 0.12	0.6 ± 0.13	0.5 ± 0.11
	High (> 5)	49 ± 6.11	3 ± 0.79	2.9 ± 0.45	0.2 ± 0.05
	MWU (p-value)	**834 (<0.000)**	**2146 (0.003)**	**1164 (<0.000)**	2885 (0.414)
Forest range area (km^2)	Low (< 80)	28.8 ± 3.65	1.6 ± 0.54	1.6 ± 0.23	0.3 ± 0.1
	High (> 80)	22.9 ± 7.28	1.4 ± 0.45	1.5 ± 0.46	0.4 ± 0.1
	MWU (p-value)	**2934 (0.005)**	**2703 (0.054)**	**2101 (0.040)**	2345 (0.186)
Cattle grazing	0	3.5 ± 1.81	0.17 ± 0.17	0.3 ± 0.18	0.1 ± 0.08
	1	18.9 ± 3.44	2 ± 0.53	1.5 ± 0.28	0.2 ± 0.05
	2	33.8 ± 4.81	0.7 ± 0.24	1.9 ± 0.44	0.9 ± 0.2
	KWH (p-value)	**9 (0.020)**	2 (0.371)	**7 (0.026)**	**14 (<0.000)**
Fire-wood collection	Low	-	-	1 ± 0.13	0.3 ± 0.07
	High	-	-	3.4 ± 0.76	0.7 ± 0.2
	MWU (p-value)	-	-	**1465 (0.006)**	**1662 (0.019)**
Disturbance from local people and tourist	0	22.6 ± 4.3	1.5 ± 0.52	0.9 ± 0.15	0.2 ± 0.06
	1	26.2 ± 3.77	0.7 ± 0.24	1.8 ± 0.36	0.5 ± 0.14
	2	64 ± 21.04	5.2 ± 1.9	5.3 ± 1.64	1.1 ± 0.38
	KWH (p-value)	3 (0.192)	**7 (0.035)**	**17 (<0.000)**	**12 (0.002)**
Study years	2018	17.9 ± 5.62	1.1 ± 0.74	1.4 ± 0.52	0.3 ± 0.11
	2019	27.8 ± 8.66	0.4 ± 0.28	1.9 ± 0.7	0.5 ± 0.18
	2020	20.2 ± 6.12	2 ± 1.26	1.6 ± 0.55	0.5 ± 0.26
	2021	22.5 ± 5.85	2.1 ± 1.02	1.3 ± 0.36	0.4 ± 0.16
	2022	32.8 ± 10.23	1.1 ± 0.76	0.9 ± 0.34	0.4 ± 0.19
	2023	41.7 ± 10.51	2.3 ± 0.74	2.2 ± 0.68	0.1 ± 0.06
	KWH (p-value)	3 (0.685)	**16 (0.005)**	4 (0.517)	5 (0.414)
Crop damage incidences	Low (< 100)	-	0.7 ± 0.18	1.3 ± 0.18	0.4 ± 0.08
	High (> 100)	-	10.8 ± 3.11	5.2 ± 1.61	0.39 ± 0.12
	MWU (p-value)	-	**276 (<0.000)**	**353 (0.001)**	813 (0.632)
Property damage incidences	Low (< 10)	-	-	1.3 ± 0.19	0.39 ± 0.07
	High (> 10)	-	-	6.9 ± 1.87	-
	MWU (p-value)	-	-	**99 (<0.000)**	619 (0.148)

S-Figure 2: Crop damage in relation grass biomass-index and perimeter of forest range (km) recorded in Nilgiri Biosphere Reserve, Western Ghats, India.

Grass biomass		
**Crop damage by elephant**	**Property damage by elephant**	**Human casualty by elephant**
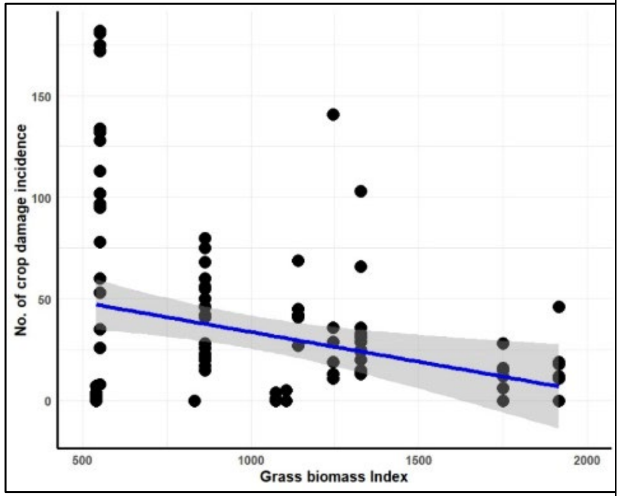	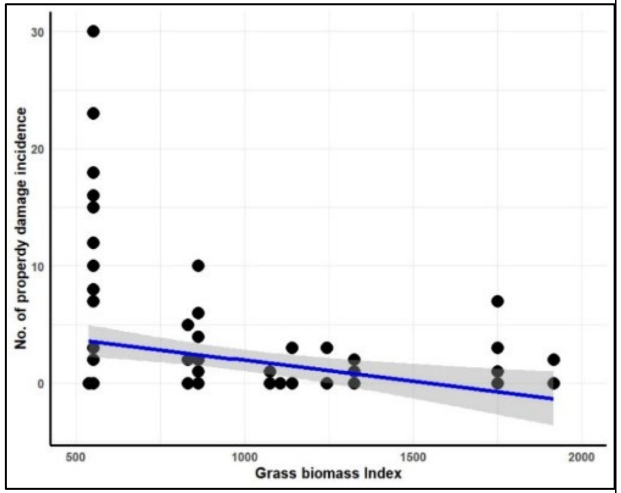	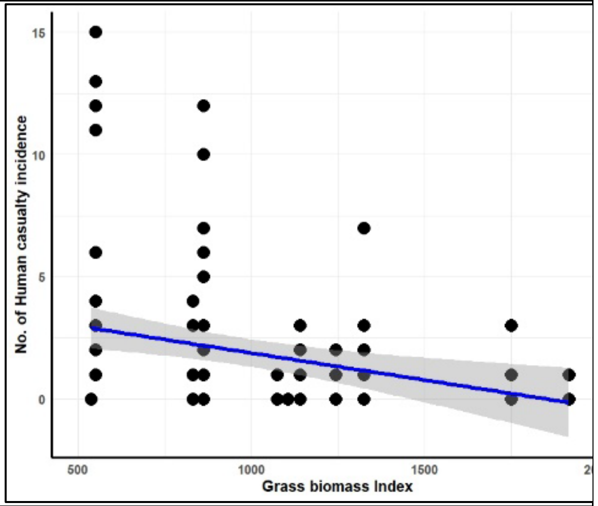
ß± SE = -0.3±0.004,<br>*f* = 29.17, *p* = 0.000; R^2= 0.59	ß± SE = -0.002±0.001,<br>*f* = 15.53, *p* = 0.000; R^2= 0.47	ß± SE = -0.001±0.000,<br>*f* = 16.66, *p* = 0.000; R^2= 0.49
**Perimeter of range**		
**Crop damage by elephant**	**Property damage by elephant**	**Human casualty by elephant**
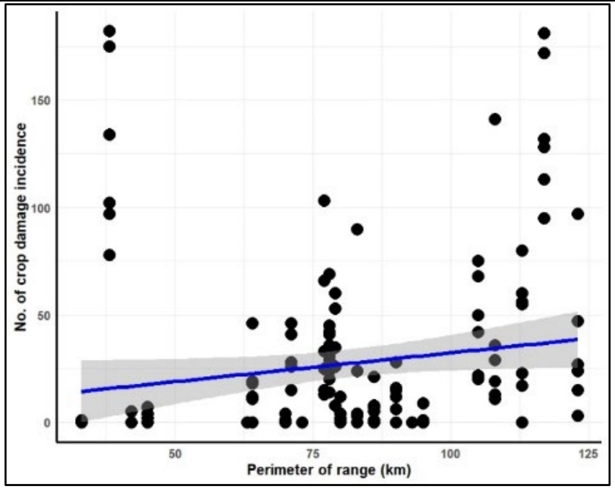	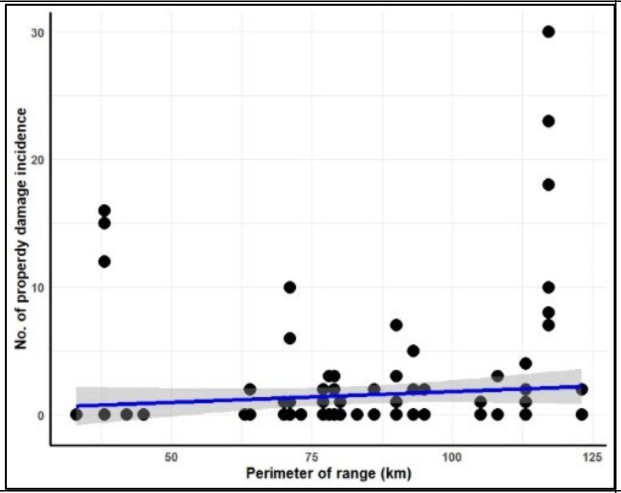	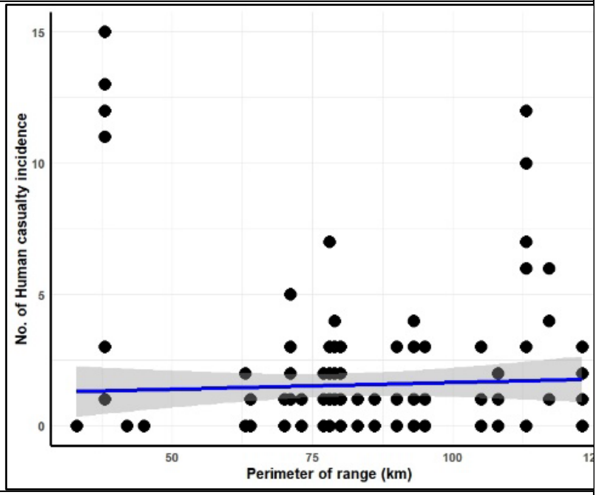
ß± SE = 0.79±0.123,<br>*f* = 29.17, *p* = 0.000; R^2= 0.59	ß± SE = 0.08±0.018,<br>*f* = 15.53, *p* = 0.000; R^2= 0.47	ß± SE = 0.064±0.000,<br>*f* = 16.66, *p* = 0.000; R^2= 0.49
